# Using a dual antibody point-of-care test with visual and digital reads to diagnose syphilis among people living with HIV in Botswana

**DOI:** 10.1177/0956462420975639

**Published:** 2021-02-11

**Authors:** Irfaan Maan, David S Lawrence, Nametso Tlhako, Kehumile Ramontshonyana, Aamirah Mussa, Adriane Wynn, Michael Marks, Doreen Ramogola-Masire, Chelsea Morroni

**Affiliations:** 1 Department of Clinical Research, Faculty of Infectious and Tropical Diseases, 218289 London School of Hygiene and Tropical Medicine, London, UK; 2 292006 Botswana Harvard AIDS Institute Partnership, Gaborone, Botswana; 3 Botswana University of Pennsylvania Partnership, Gaborone, Botswana; 4 Division of Infectious Diseases & Global Public Health, 8784 University of California, San Diego, USA; 5 Hospital for Tropical Diseases, London, UK; 6 54547 University of Botswana, Gaborone, Botswana; 7 Department of International Public Health, 9655 Liverpool School of Tropical Medicine, Liverpool, UK

**Keywords:** Syphilis, HIV, sexually transmitted infections, point-of-care test, Botswana, Africa

## Abstract

Syphilis data from low- and middle-income countries are lacking due to limited testing. Point-of-care tests (POCTs) have been promoted to expand testing but previously only included treponemal tests, which cannot distinguish active from past infection. We aimed to assess the feasibility of using a combined treponemal and non-treponemal POCT in HIV clinic patients in Gaborone, Botswana, and estimate syphilis prevalence in our clinic sample using this approach. We recruited 390 non-pregnant patients. Participants underwent a combined treponemal and non-treponemal POCT (Dual Path Platform (DPP®) Syphilis Screen and Confirm Assay (Chembio Diagnostic Systems)) on finger-prick blood sample and a questionnaire. Median age 45 years, 30% men, median CD4 count 565 cells/μL, and 91% had an HIV viral load <400 copies/mL. Five participants had active syphilis (1.3%, 95% CI 0.5–3.0%) and 64 had previous syphilis (16.4%, 95% CI 13.0–20.4%) using the DPP POCT. There was a reasonable level of agreement between digital and visual reading of the POCT (kappa statistic of 0.81); however, visual reading missed three active infections (60%). The level of active syphilis was similar to local antenatal data. The DPP POCT led to five participants with active syphilis being diagnosed and starting same-day treatment. The digital reader should be used.

## Introduction

An estimated six million new cases of syphilis occur worldwide each year, with the highest incidence and prevalence in Africa.^1,2^ In low- and middle-income countries (LMICs), syphilis prevalence estimates are predominantly based on routine antenatal care (ANC) data, which are extrapolated to the general population.^3^ Antenatal care screening is prioritized to avert cases of mother-to-child transmission of syphilis which is associated with stillbirth and other poor outcomes.^4,5^ Syphilis also increases the risk of HIV transmission and acquisition, and syphilis infection may lead to more severe disease manifestations, including neurosyphilis, in those living with HIV.^6^ The effective diagnosis and treatment of syphilis among high-risk groups, including people living with HIV (PLHIV), is important.

Most syphilis prevalence data from Botswana are from ANC, last reported in 2009, to be 1.3% nationally and 0.8% in Gaborone, the capital city.^7^ An analysis of antenatal HIV and rapid plasma reagin (RPR) test results from different clinics in Botswana, across various periods between 2008 and 2016, found HIV-positive women to have a syphilis infection rate of 1.5% and that HIV-positive women were more likely to be infected with syphilis than HIV-negative women.^8^ A 2012 study of at-risk groups in three urban areas in Botswana found syphilis prevalence to be 3.5% among female commercial sex workers (CSWs) and 2.7% among men who have sex with men (MSM).^9,10^ The current adult HIV prevalence in Botswana is 20.3%.^11^ A study assessing syphilis prevalence among PLHIV conducted at the country’s largest specialist HIV clinic in 2002, the same setting as the current study, tested 143 patients at antiretroviral treatment (ART) initiation and found 9.8% with a positive non-treponemal RPR test and a positive treponemal hemagglutination (TPHA) test.^12^ That study was conducted prior to the implementation of the Botswana national ART programme in 2002; as of 2018, ART coverage of eligible PLHIV was 74–83% in Botswana.^11,13^

Where diagnostic access is limited, as is the case in Botswana, a syndromic approach to genital ulcer disease (GUD) is usually adopted. Patients with GUD are treated with Benzathine Penicillin G and ceftriaxone to cover for both syphilis and chancroid.^14^ Syndromic management has limitations including the potential for unnecessary antimicrobial prescribing, only detecting symptomatic patients, and not reaching a definitive diagnosis.^14,15^ The World Health Organization (WHO) has advocated that countries begin to move away from syndromic management as part of the global strategy for sexually transmitted infection (STI) control.^16^ A recent trial in Rwanda demonstrated that introducing point-of-care tests (POCTs), including for syphilis, allowed more accurate and targeted treatment of STIs.^17^ This strategy has the potential to greatly improve the delivery of care for patients with STIs in LMICs. The WHO has advocated the use of syphilis POCTs to expand the availability of testing and reduce loss to follow-up and has added such tests to its list of prequalified diagnostics.^16,18^

Earlier syphilis POCTs detected only treponemal antibodies and therefore could not distinguish between active syphilis infection (i.e. a positive treponemal and non-treponemal test) and past exposure to treponemal infection (i.e. a positive treponemal test only). More recent tests such as the Dual Path Platform (DPP®) Syphilis Screen and Confirm Assay (Chembio Diagnostic Systems, New York) (DPP POCT) are different in that they incorporate both a treponemal and non-treponemal test (reactive at RPR ≥ 1:2) that can be used on whole blood, plasma or serum.^19^ These tests, which currently cost approximately US$2.50 per test, can be read manually by a healthcare worker or analyzed using a digital reader.^20^ A 2016 meta-analysis of published and unpublished data using the DPP syphilis POCT collated nine evaluations representing more than 7200 individual samples.^21^ Overall agreement between the DPP POCT and reference serology (RPR, Venereal Disease Research Laboratory, treponema pallidum particle agglutination or TPHA) was 85.2%. High titre samples (RPR ≥ 1:16) had a 98% sensitivity and 100% specificity for the treponemal test and 98% sensitivity for the non-treponemal test. Low titre samples (RPR < 1:16) had a 93% sensitivity and 98.5% specificity for the treponemal test and 85% sensitivity and 87.6% specificity for the non–treponemal test.^21^ A 2017 field evaluation of the DPP POCT visual reading compared to laboratory tests found misclassification of the non-treponemal test with RPR titres <1:8.^22^ Tests conducted by the manufacturer of DPP indicate there is a correlation between RPR titre and density of the non-treponemal test line and that digital readers were better at reading low RPR titres than a visual read.^19^ The use of a digital reader for DPP may therefore increase its sensitivity.

We performed a study to assess the feasibility of using a combined treponemal and non-treponemal POCT in patients attending an HIV clinic in Botswana and compared the outcomes of visual versus digital readings of the POCT result. We also estimated the prevalence of syphilis infection using this approach in non-pregnant adults attending our clinic.

## Methods

### Study design, setting and sample

We conducted a cross-sectional study in the Infectious Disease Care Clinic (IDCC) at Princess Marina Hospital (PMH), the largest public referral hospital in Botswana. The IDCC is an outpatient clinic which provides care for PLHIV from Gaborone and outlying areas. We approached all consecutive clinic attendees aged 18 years or over presenting for routine appointments, every day of clinic operation, over a four-week period. Patients were ineligible if they were unable to consent, currently receiving treatment for syphilis (so as not to unduly burden the patient), or pregnant (all pregnant women are routinely tested at their ANC booking visit and treated as per Botswana national guidelines). Using syphilis prevalence data from CSWs, MSM and pregnant women in Botswana, the prevalence among HIV patients in Botswana was estimated at 2%. We calculated that a sample size of 335 was needed to measure a prevalence of 2 ± 1.5%.

### Study procedures

All IDCC clinic patients were briefly informed about the study in Setswana, the local language, and English, as they registered for their HIV appointment; more detailed announcements were made outside clinic rooms whilst patients waited to see a doctor. Those who were interested in participating were provided with additional information by study staff. After screening and written consent, eligible participants were enrolled.

Data were collected on socio-demographic characteristics, medical history, sexual health behaviours and history of syphilis and GUD. Interviews were conducted in the participant’s language of preference by a research assistant. HIV viral load (VL), CD4 cell count, ART status and previous RPR tests (the standard syphilis screening test used at HIV diagnosis and whenever clinically indicated) were extracted from medical notes. The DPP POCT was conducted by research nurses, who were trained in the test procedures. All tests were conducted by the two nurses, in rotation, throughout the study period.

**Figure 1.**
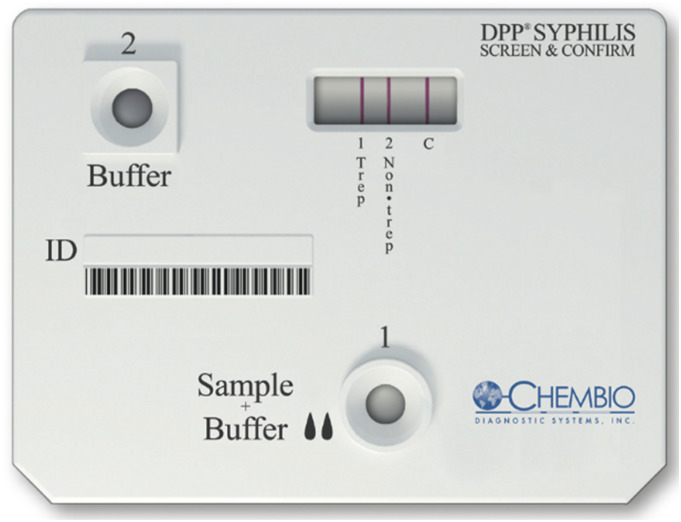
Dual Path Platform Syphilis Screen and Confirm Assay (Chembio Diagnostic Systems, New York); complete cassette.

The POCT (Figure 1) has three lines, a treponemal, a non-treponemal and a control line (which indicates whether the test is functioning). The test requires 10 μL of blood, and pipettes are included in the kit. The blood is added to the first well along with two drops of a buffer solution. After five minutes, five drops of buffer solution are added to the second well, and the test is read after a further 15 min. A visible line indicates a reactive test at the treponemal, non-treponemal and control lines. If no control line was visible, the test was considered unusable and was repeated.

All POCT results were first read visually and recorded by the nurse, and then separately read by the study doctor, blinded to visual result, using a battery-powered digital reader, which gives a positive or negative result for each test line. The reader was handheld, similar in size to the POCT, clipped onto the test cassette and operated by pressing a single button. The digital read was considered the definitive result on which treatment decisions were based, in line with the manufacturer’s instructions.^19^

Point-of-care tests results were given to all participants at the same clinic visited by the research team and recorded in the participant’s medical notes. All participants with active syphilis (reactive treponemal and non-treponemal tests) underwent a clinical assessment to identify neurological symptoms that might warrant further investigation and were prescribed doxycycline (100 mg twice a day for 28 days), in line with the Botswana national STI treatment guidelines and current drug availability, due to stock outs of Benzathine Penicillin G. They were also counselled about partner notification and given contact slips, the standard for STI partner notification in Botswana. Participants with a positive treponemal test were counselled on past syphilis infection. All participants were asked a single open-ended question about whether they would want to take part in future STI screening and treatment in the context of their HIV care.

### Statistical analysis

The primary outcome was active syphilis infection, defined as a positive treponemal and non-treponemal antibody result. Secondary outcomes were proportion of participants testing positive for treponemal antibody alone (past syphilis infection) and non-treponemal antibody alone (non-specific reactive result). We compared the results of visual versus digital read of the POCT. Descriptive data were generated for active syphilis infection and participant characteristics. Bivariate associations of participant characteristics with active syphilis and past syphilis infection were evaluated using chi-squared and Fisher’s exact tests and unadjusted odds ratios. Agreement between visual and digital POCT readings was calculated using an unweighted kappa statistic. The sensitivity and specificity of visual readings compared to the definitive digital reading results were calculated for active syphilis (versus other) and past syphilis infection (versus other). Paper questionnaires were double-entered into EpiData v4.2 and analyzed using Stata 14.

### Ethical approval

The protocol was approved by the Botswana Health Research Development Committee at the Ministry of Health and Wellness, the University of Botswana Office of Research and Development Research Ethics Committee, the Research and Ethics Committee of PMH and the London School of Hygiene and Tropical Medicine MSc Research Ethics Committee. We obtained written consent from all participants.

## Results

### Sample characteristics

**Figure 2.**
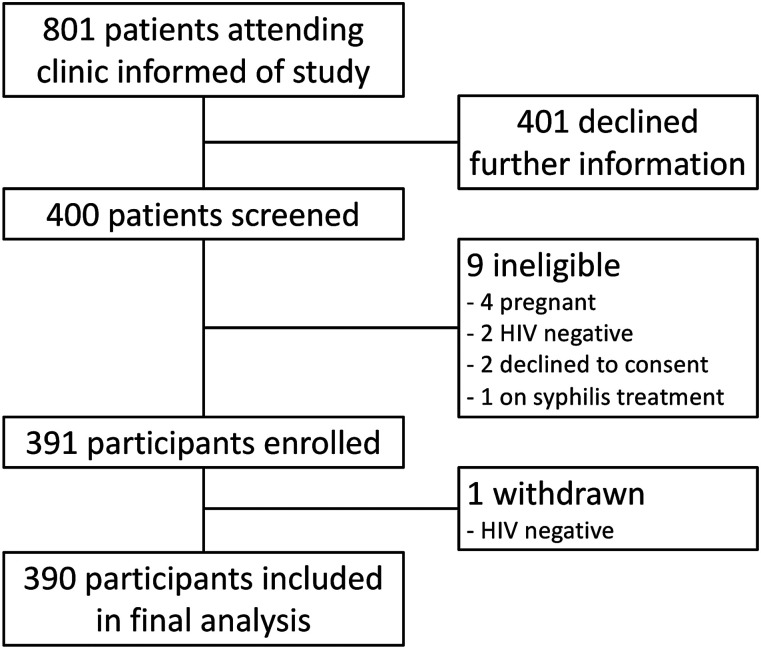
Flow of participants in a study of syphilis testing, using Dual Path Platform Point-of-care tests during their HIV care clinic visit in Botswana.

During the four-week study period from July to August 2017, 801 adult patients attended the HIV clinic of whom 301 were men (38%) and 500 women (62%). A total of 400 attendees (50%) expressed interest in study participation and were screened for eligibility; 391 (98%) were enrolled, and 390 included in analysis (Figure 2).

Seventy percent (*n* = 272) were women and 30% (*n* = 118) were men (Table 1). Median age was 45 years (interquartile range (IQR) 40–51 years), and median CD4 cell count was 565 cells/μL (IQR 392–729 cells/μL). Three hundred and eighty-seven participants (99%) were on ART and 344 (91%) had a VL < 400 copies/mL. Seventy-three participants (19%) reported having had a syphilis test in the past, with 11 of those (15%) reporting a previous positive syphilis test result (five men and six women). No participants reported current genital ulcers. Ninety-nine participants (25.5%) reported having had genital ulcers in the past, of whom 23 reported them as painless (6% of all participants).

**Table 1.** Socio-demographic and health history characteristics of non-pregnant HIV clinic attendees in Botswana participating in a syphilis testing study using DPP POCT during an HIV care clinic visit.

Characteristic	*N* = 390 *n* (%)
Gender (*n* = 390)	
Male	118 (30%)
Female	272 (70%)
Age (*n* = 390); median age in years (IQR)	45 (40–51)
20–29 years	8 (2%)
30–39 years	72 (18%)
40–49 years	187 (48%)
50 years or older	123 (31%)
Relationship status (*n* = 389)	
Married	109 (28%)
Current relationship, not married	178 (46%)
Single, no current relationship	102 (26%)
Education (*n* = 379)	
None	33 (9%)
Primary	100 (26%)
Secondary	186 (49%)
Higher than secondary/vocational	60 (16%)
CD4 cells/μL (*n* = 380); median CD4 cells/μL (IQR)	565 (392–729)
<200	25 (7%)
200–349	49 (13%)
350–500	80 (21%)
>500	226 (59%)
Viral load, copies/mL <400 (*n* = 376)	344 (91%)
Number of sexual partners in past 12 months (*n* = 389)	
None	76 (19.5%)
One	282 (72.5%)
Two or more	31 (8%)
Condom used at last sexual intercourse (*n* = 380)	266 (70%)
Men who have sex with men (*n* = 357)	5 (1.4%)
Reports having had a previous syphilis test (*n* = 386)	73 (19%)
Previous syphilis test result positive (*n* = 73)	11 (15%)
Reports history of genital ulcers (*n* = 388)	99 (25.5%)
Reports history of painless genital ulcers (*n* = 388)	23 (6%)

DPP®: Dual Path Platform; POCT: Point-of-care test; IQR: interquartile range.

### Point-of-care tests outcomes

All finger-prick blood samples produced usable POCT results as indicated by a positive control line. The median time from sample collection to reading of the result was 20 min (range 18–30 min). Results of the DPP POCTs are presented in Table 2. Using the definitive digital reading results, five of the 390 participants had both positive treponemal and non-treponemal test results (1.3%, 95% CI 0.5–3.0%), indicating active syphilis infection; all five initiated syphilis treatment at the same clinic visit. Sixty-four of the 390 participants tested positive for treponemal antibody alone (16.4%, 95% CI 13.0–20.4%) indicating evidence of a past syphilis infection. Ten participants tested positive for the non-treponemal test alone (2.6%; 95% CI 1.4–4.7%), indicating a non-specific reactive result. The total number of participants with syphilis exposure (combining active and past syphilis infection) was 69 (17.7%; 95% CI 14.2–21.8%).

**Table 2.** Results of syphilis testing using the DPP POCT (with digital reading) among non-pregnant HIV clinic attendees participating in a syphilis testing study during an HIV care clinic visit in Botswana.

Result	Number (*n* = 390)	Prevalence in our clinic sample, %	95% confidence interval
Active syphilis infection (treponemal and non-treponemal)	5	1.3	0.5–3.0%
Past syphilis infection (treponemal only)	64	16.4	13.0–20.4%
Non-specific reactive result (non-treponemal only)	10	2.6	1.4–4.7%
Negative (non-reactive)	311	79.7	75.4–83.5%
Total syphilis exposure (active plus past)	69	17.7	14.2–21.8%

DPP®: Dual Path Platform; POCT: Point-of-care test.

Of the 23 participants who reported ever having painless genital ulcers in the past, 35% (*n* = 8) tested positive for the treponemal antibody. The majority of the 69 participants who tested positive for treponemal antibody (*n* = 54, 78%) did not report any history of genital ulceration, painless or painful. Of the 11 participants who reported having had a positive syphilis test result in the past, nine had negative test results, one tested positive for treponemal antibody alone and one tested positive for the non-treponemal test alone.

In bivariate analysis, no characteristics were associated with active syphilis infection (data not shown). The only characteristics associated with active or past syphilis infection were increasing age (*p* < 0.001) and lower levels of education (*p* = 0.001). There was no association between reporting a history of genital ulceration (any, painless or painful) with syphilis infection or treponemal exposure. Five (1.4%) of the participants identified as MSM; all of whom had negative test results.

### Performance of visual versus digital readings

Overall agreement between visual and digital readings was 93.85%, giving a kappa statistic of 0.812 (Table 3). Using the digital reading as the definitive result, visual reading misclassified three of the five (60%) active syphilis infections as past syphilis infections. For active syphilis (both positive treponemal and non-treponemal tests), the sensitivity of the visual reading compared to the definitive digital reading was 40% (95% CI 5.27–85.3%) and the specificity was 99.5% (95% CI 98.1–99.9%). For treponemal antibody alone (indicating prior syphilis infection), the sensitivity of visual reading compared to definitive digital reading was 89.1% (95% CI 78.8–95.5%) and the specificity was 96.9% (95% CI 94.4–98.5%).

**Table 3.** Diagnostic performance of digital versus visual reading of the DPP® Syphilis Screen and Confirm Assay among non-pregnant HIV clinic attendees participating in a syphilis testing study during an HIV care clinic visit in Botswana.

A. Agreement between digital and visual reading of DPP® sSyphilis Screen and Confirm Assay									
		Digital reading							
		Active syphilis infection	Past syphilis infection		Non-specific reactive result		Negative	Total	
Visual reading									
Active syphilis infection		2	2		0		0	4	
Past syphilis infection		3	57		1		6	67	
Non-specific reactive result		0	0		3		1	4	
Negative		0	5		6		304	315	
Total		5	64		10		311	390	
B. Agreement between digital and visual reading of DPP® Syphilis Screen and Confirm Assay (Cohen’s kappa statistic)									
Expected agreement	Observed agreement			Kappa		Standard error			*p*-value
67.27%	93.85%			0.812		0.04			<0.0001
C. Sensitivity, specificity, positive and negative predictive value for *active syphilis infection* of the visual versus digital (as reference standard) reading of DPP® Syphilis Screen and Confirm Assay									
				Visual +ve		Visual −ve			Total
		Digital +ve		2		3			5
		Digital −ve		2		383			385
		Total		4		386			390
Sensitivity				40%		95% CI 5.27%–85.3%			
Specificity				99.5%		95% CI 98.1%–99.9%			
Positive predictive value				50.0%		95% CI 6.76%–93.2%			
Negative predictive value				99.2%		95% CI 97.7%–99.8%			
D. Sensitivity, specificity, positive, and negative predictive value for *past syphilis infection* of the visual versus digital (as reference standard) reading of DPP® Syphilis Screen and Confirm Assay									
				Visual +ve		Visual −ve			Total
		Digital +ve		57		7			64
		Digital −ve		10		316			326
		Total		67		323			390
Sensitivity				89.1%		95% CI 78.8–95.5%			
Specificity				96.9%		95% CI 94.4–98.5%			
Positive predictive value				85.1%		95% CI 74.3–92.6%			
Negative predictive value				97.8%		95% CI 95.6–99.1%			

DPP®: Dual Path Platform; CI: confidence interval.

Almost all participants (96%, *n* = 365) reported that they would like to participate in STI testing programmes in the context of their HIV care in the future.

## Discussion

In this cross-sectional study of self-selected non-pregnant patients attending an HIV clinic in Gaborone, Botswana, the prevalence of active syphilis infection was 1.3% using the DPP POCT. This is the second study to investigate syphilis prevalence among HIV-infected individuals in Botswana, and the first in the era of ART, using a POCT to test and treat on the same day. All DPP POCTs gave usable results, shown by a visible control line, and led to the five participants with a positive result for active syphilis infection being diagnosed and starting treatment on the same day.

The proportion of participants in this study sample with active syphilis infection is comparable to the 1.6% prevalence across the WHO African region and 0.8% prevalence among Gaborone ANC attendees,^3,7^ but substantially lower than was found in a study conducted in 2002 in the same HIV clinic, which estimated a syphilis prevalence of 9.8%.^12^ The lower proportion of syphilis infection found in our study compared to the earlier study in the HIV clinic population may reflect a reduction in syphilis in the overall population, a general trend that has also been observed in Botswana’s ANC data between 2004 and 2008 and which is attributed to syndromic STI management.^23^ However, it is also likely to represent differences in the study populations which were independently selected from the clinic. In the earlier study, all participants were ART-naïve, with a median CD4 cell count of 104 cell/μL and had a lower median age than our population of older, ART-experienced individuals, and no exclusion criteria were noted. The estimated level of active syphilis in this group of ART-experienced, non-pregnant HIV clinic attendees was lower than in other at-risk groups such as MSM and CSWs in Botswana.^9,10,16^ Very few MSMs were included in our sample, and no data on CSWs were collected, precluding any robust estimates in these groups.

Although no participant characteristics were associated with active syphilis infection in this small study, we identified some factors associated with past infection. Increasing age was associated with syphilis exposure with the proportion increasing from 6% in those aged 20–30 years to 28% in those over 50 years. This is similar to the findings of a population-based survey in Rwanda^24^ and a study conducted in Ethiopia^25^ and likely reflects the longer duration of exposure risk with advancing age and measurement of an antibody which persists for life. Lower education levels were associated with higher rates of syphilis exposure, which has also been reported in prior African studies^25^ but may also reflect an association between increasing age and lower educational attainment. Most people reporting a history of genital ulceration in our study did not test positive for treponemal antibody suggesting a different pathology of GUD in the majority of cases in our setting. Although painless genital ulceration was associated with the presence of treponemal antibody, the majority of those with treponemal antibody reported no history of genital ulceration. This highlights the importance of moving towards diagnostic-driven diagnosis for STIs using rapid diagnostics such as the DPP POCTs in resource-constrained environments.

There was a high level of agreement between digital and visual reading of the DPP POCT in this study, and the kappa statistic of 0.81 indicates good agreement beyond chance. However, visual reading missed three of the five active infections (60%). Our data therefore suggest that the digital reader should be used when performing the DPP POCT to avoid missing active infections. No previous published data have compared visual and digital readings for the DPP POCT; one previous laboratory-based study looking at the DPP POCT found a 1.5% disagreement between two visual operators.^26^ If digital readers are not available, a visual reading by two individuals trained in use of the POCTs should be undertaken as this has been shown in field tests to have 99.5% agreement.^22^

Using the dual antibody DPP test, compared to the results that would have been provided by RPR screening alone, prevented 10 patients with non-specific reactive results in this study from unnecessarily being treated for syphilis. Importantly, compared to a treponemal-only POCT, the 64 treponemal antibody-only participants were not treated or required to undergo further blood tests. In practice, using the dual antibody test and correlating with any previous treatment history could reduce the number of cases misdiagnosed as active syphilis, obviating the need for treatment and contact tracing. This would avoid causing unnecessary distress for patients and conserve time and resources for healthcare services which are particularly important, given periods of global Benzathine Penicillin G shortage.^27,28^ The current price of the DPP POCT at US$2.50 is significantly more expensive than the average cost per vial of Benzathine Penicillin G in LMICs at US$0.20.^28^ However, this comparison does not take into account the clinic and staff costs associated with its administration or the implications of exacerbating present drug shortages. Detailed costing studies are required to further evaluate the utility of this approach.

Our study has several limitations including the relatively small sample size, clinic-based recruitment, the self-selection of participants into the study and the exclusion of pregnant women and patients currently being treated for syphilis, limiting the representativeness of our sample of the HIV-positive population of Botswana and our ability to generalize the prevalence of active syphilis to the population of PLHIV in Botswana. In addition, 62% of clinic attendees but 70% of the included population were women. Participants were actively engaged with health services and on ART with high CD4 cell counts and suppressed HIV VLs, which could potentially exclude those most at risk of active syphilis. However, the study population is representative of approximately 70% of the PLHIV in Botswana.^13^ Medical notes were often incomplete with respect to previous syphilis test data so that information was self-reported. A comparator laboratory test was not performed to confirm the results. The DPP POCT is less sensitive in those with an RPR titre <1:16, and patients with advanced immunosuppression often do not develop high RPR titre response to syphilis infection; and individuals with late latent syphilis may have been missed. Data from the manufacturer indicate a correlation between the density of the non-treponemal line displayed on the digital readers and RPR titres; however, these data were not captured in this study.^19^ It was therefore not always possible to determine if patients with a positive treponemal antibody alone had either previously treated syphilis or late latent syphilis. A cost-benefit analysis was not included in this study. A previous study found the dual antibody POCT, at its current price, was less cost-effective in two Sub-Saharan African sites compared to treponemal antibody tests and would need to be reduced in price by a half to be cost-effective in those settings.^20^ Only half of clinic attendees over the study period participated and reasons for declining participation were not collected.

In conclusion, the results of our study show the level of active syphilis in this clinic-based sample of PLHIV to be similar to national ANC prevalence and lower than in other at-risk groups in Botswana. Testing with the DPP POCT, using a digital reader, was able to give same-day results to participants during a clinic visit, differentiate between active and past infection, identify five active infections requiring treatment and reduce unnecessary antimicrobial use. The digital reader should be used when using the DPP POCT to avoid missing infections.
